# Educational level and cardiovascular outcomes in patients hospitalized with first-detected atrial fibrillation: a Swedish nationwide cohort study

**DOI:** 10.1186/s12872-026-06095-y

**Published:** 2026-07-24

**Authors:** Áron Sztaniszláv, Anna Björkenheim, Anders Magnuson, Nils Edvardsson, Dritan Poçi

**Affiliations:** 1 Department of Cardiology, Faculty of Medicine and Health, Örebro University, Örebro, Sweden; 2 Faculty of Medicine and Health, Clinical Epidemiology and Biostatistics, School of Medical Sciences, Örebro University, Örebro, Sweden; 3 Department of Molecular and Clinical Medicine, Institute of Medicine, Sahlgrenska Academy, Gothenburg, Sweden; 4 Department of Clinical Physiology, Sahlgrenska University Hospital, Gothenburg, Sweden; 5 Faculty of Medicine and Health, Örebro University, Örebro, Sweden

**Keywords:** Atrial fibrillation, Morbidity, Socioeconomic status, Education

## Abstract

**Background:**

Socioeconomic factors are associated with cardiovascular morbidity. We investigated the association between education level and incident heart failure (HF), acute coronary events (AMI), and cerebrovascular events (stroke) following hospitalization with first-detected atrial fibrillation (AF).

**Methods:**

In this nationwide retrospective observational cohort study using linked Swedish national registers, we included patients who received a first diagnosis of AF during hospitalization from 1995 to 2008. Education levels were categorized as primary, secondary, or academic, and patients were followed for up to five years. Outcomes were first hospitalization for HF, AMI, or stroke. Associations were assessed using cause-specific Cox proportional hazard models, for men and women separately. Models were adjusted for age, calendar year of AF diagnosis, whether AF was the primary or secondary admission diagnosis, comorbidity diagnoses and thromboembolic risk.

**Results:**

The cohort comprised 263,172 patients (mean age 72.5 ± 10.4 years; 56.2% male). Compared with primary education, secondary and academic education levels were associated with lower adjusted risks of HF and AMI in both females and males. For HF, adjusted hazard ratios (HR) were 0.96 (95% CI 0.93–1.00) for secondary and 0.82 (95% CI 0.77–0.87) for academic education in females, and 0.93 (95% CI 0.90–0.96) and 0.76 (95% CI 0.72–0.80), respectively, in males. For AMI, adjusted HRs were 0.89 (95% CI 0.85–0.93) and 0.71 (95% CI 0.65–0.77) in females, and 0.91 (95% CI 0.87–0.94) and 0.75 (95% CI 0.71–0.80) in males. For stroke, lower adjusted risks were observed only in the academic education group. Baseline comorbidity burden and thromboembolic risk were higher in lower education groups.

**Conclusions:**

In this large nationwide cohort of patients hospitalized with AF, higher education level was associated with lower adjusted hazards of incident HF and AMI over five years, while the association with stroke was weaker. Education level may be considered a risk marker when planning follow-up and preventive care after hospitalization with AF.

**Supplementary Information:**

The online version contains supplementary material available at https://doi.org/10.1186/s12872-026-06095-y.

## Introduction

Education level is an important indicator of socioeconomic status (SES) and is associated with health literacy, long-term income, healthcare-seeking behaviour, and the ability to navigate healthcare systems. Socioeconomic status, in turn, is associated with the incidence, prevalence, and outcomes of cardiovascular diseases [1–3]. These associations may reflect differences in lifestyle-related risk factors, comorbidity burden, access to preventive care, treatment initiation, and adherence to long-term therapy [4, 5]. 

Atrial fibrillation (AF) is associated with substantial morbidity and mortality and imposes a considerable economic burden, largely driven by hospitalization [6, 7]. Patients hospitalized with AF are at risk of subsequent cardiovascular events, including heart failure (HF), acute myocardial infarction, and stroke [8]. Although hospitalization rates for AF increased during the 1980s and 1990s, more recent data suggest a declining trend in inpatient treatment [9, 10]. 

In a previous study, we found an inverse association between education level and all-cause mortality among patients hospitalized with a first-detected AF [11]. While mortality is an important endpoint, morbidity outcomes may provide additional insight into the clinical course after the hospitalization with AF. Morbidity data are also relevant for evaluating healthcare needs, planning follow-up, and identifying patients who may benefit from optimized preventive care [12]. 

Associations between SES, including education level, and cardiovascular morbidity have been described in the general population [1, 13, 14]. However, evidence on whether education level is associated with subsequent cardiovascular outcomes among patients hospitalized with AF remains limited.

The aim of this nationwide retrospective observational cohort study was to investigate the association between education level and subsequent first hospitalizations for heart failure, acute coronary events (AMI), and cerebrovascular events (stroke) during five years of follow-up after a first detected AF diagnosis during hospitalization in Sweden.

## Methods

### Patient population

This study was based on a previously described patient population [15, 16]. We identified all individuals with a diagnosis of AF first recorded while hospitalized in Sweden from January 1, 1995, through December 31, 2008. Data were obtained by cross-linking the Swedish National Patient Registry and the Swedish Cause of Death Registry administered by the Swedish National Board of Health and Welfare, with the Total Population Register from Statistics Sweden.

Sweden has a publicly funded healthcare system and high-quality nationwide registries; the accuracy and validity of these registries have been evaluated previously [17, 18]. Diagnoses were coded according to the International Classification of Diseases (ICD) using ICD-9 from 1987 to 1996 and ICD-10 beginning in 1997. The ICD codes for AF and study outcomes were defined as in previous studies [15, 16]. We did not differentiate among paroxysmal AF, persistent AF, permanent AF, and atrial flutter.

### Study design

This nationwide register-based observational cohort study identified all individuals with an AF diagnosis first recorded during hospitalization. Exclusion criteria were: unavailable education level, age less than 30 years, age more than 85 years at AF diagnosis, and date of AF diagnosis before 1995. Patients younger than 30 years were excluded to ensure reliable classification of the highest education level attained. Patients older than 85 years were excluded, consistent with the previously described cohort design [15]. This was due to missing data in this age group and an insufficient number of controls in the previous cohort construction. Morbidity endpoint data were available through December 31, 2009.

We categorized the cohort into three groups based on highest education level attained according to the International Standard Classification of Education (ISCED): primary (ISCED 1–2), secondary (ISCED 3–4), and academic (ISCED 5–8) education [19, 20]. This grouping was chosen to reflect commonly used and clinically interpretable levels of education while preserving sufficient sample size in each category for sex-stratified analyses. Outcomes were a first hospitalization for heart failure, an acute coronary event, or a cerebrovascular event during follow-up. Follow-up started on the date of the index hospitalization and continued for up to five years, with censoring at death, emigration, or the end of the study period, December 31, 2009.

Patients who died within 30 days following the index hospitalization were excluded from the main survival analyses to focus on subsequent morbidity after AF hospitalization rather than events occurring during, or shortly after, the index admission. Sensitivity analyses including these patients were performed to assess the influence of this exclusion. To define incident outcomes, endpoint-specific analyses excluded patients with a record of the respective diagnosis prior to the index hospitalization, using all available National Patient Registry data from 1987 to 1994 (the earliest period with nationwide ICD-coded data). We used broader ICD-based definitions of acute coronary events (referred to as AMI) and cerebrovascular events (referred to as stroke) to reduce the risk of outcome misclassification, given the differences in diagnostic criteria and coding practices during the follow-up period. Individuals with prior HF, AMI, or stroke were excluded from the respective endpoint analyses. Comorbidity burden was characterized using the Charlson Comorbidity Index (CCI), which summarizes comorbidity-related mortality risk [21, 22]. The ICD-9 and ICD-10 codes for CCI conditions (Supplementary Table 2) were defined according to Quan et al. [23] The comorbidity burden was categorized as low (CCI 0–2), moderate (CCI 3–4), or high (CCI ≥ 5), consistent with the previously described dataset [15]. We calculated the CHA_2_DS_2_-VA score to describe thromboembolic risk [24, 25] (Table 1).

**Table 1:** Baseline characteristics of the study population by educational attainment

Variable	Education group *n* = 263 172			
	Primary	Secondary	Academic	*P* value*
*n* (%)	151 470 (57.6)	78 741 (29.9)	32 961 (12.5)	-
Females, *n* (%)	74 629 (49.3)	29 880 (37.8)	10 890 (33.0)	-
Females, *n* = 115 399				
Age, mean ± SD	76.8 ± 7.2	72.1 ± 10.1	69.0 ± 11.1	*p* < 0.001
Age categories, *n* (%)				
< 65	4 985 (6.7)	6 298 (21.1)	3 403 (31.3)	*p* < 0.001
65–74	16 657 (22.3)	8 693 (29.1)	3 447 (31.7)	
75–85	52 987 (71.0)	14 889 (49.8)	4 040 (37.1)	
Stroke risk (CHA_2_DS_2_-VA score categories), *n* (%)				
0 – Low risk	2 664 (3.6)	3 827 (12.8)	2 407 (22.1)	*p* < 0.001
1 – Moderate risk	7 942 (10.6)	5 051 (16.9)	2 386 (21.9)	
2 – OAC indication risk	5 412 (7.3)	3 180 (10.6)	1 194 (11.0)	
3 – High risk	18 699 (25.1)	6 293 (21.1)	2 033 (18.7)	
≥ 4 – Very high risk	39 912 (53.5)	11 529 (38.6)	2 870 (26.4)	
Comorbidity burden (CCI) categories, *n* (%)				
Low mortality risk (CCI 0–2)	1 562 (2.1)	2 713 (9.1)	1 741 (16.0)	*p* < 0.001
Moderate mortality risk (CCI 3–4)	22 654 (30.1)	11 581 (38.8)	4 805 (44.1)	
High mortality risk (CCI ≥ 5)	50 413 (67.6)	15 586 (52.2)	4 344 (39.9)	
Males (*n* = 147,773)	76 841	48 861	22 071	-
Age, mean ± SD	73.3 ± 9.2	68.4 ± 11.9	66.2 ± 12.1	*p* < 0.001
Age categories, *n* (%)				
< 65	12 790 (16.6)	16 461 (33.7)	9 011 (40.8)	*p* < 0.001
65–74	22 560 (29.4)	14 194 (29.1)	6 659 (30.2)	
75–85	41 491 (54.0)	18 206 (37.3)	6 401 (29.0)	
Stroke risk (CHA_2_DS_2_-VA) categories, *n* (%)				
0 – Low risk	6 108 (8.0)	9 221 (18.9)	5 779 (26.2)	*p* < 0.001
1 – Moderate risk	10 918 (14.2)	8 992 (18.4)	4 528 (20.5)	
2 – OAC indication risk	8 694 (11.3)	6 159 (12.6)	2 811 (12.7)	
3 – High risk	16 660 (21.7)	8 862 (18.1)	3 574 (16.2)	
≥ 4 – Very high risk	34 461 (44.9)	15 627 (32.0)	5 379 (24.4)	
Comorbidity burden (CCI) categories, *n* (%)				
Low mortality risk (CCI 0–2)	4 809 (6.1)	8 493 (17.4)	5 096 (23.1)	*p* < 0.001
Moderate mortality risk (CCI 3–4)	26 796 (34.9)	18 503 (37.9)	9 318 (42.2)	
High mortality risk (CCI ≥ 5)	45 236 (58.9)	21 865 (44.8)	7 657 (34.7)	

Continuous variables are presented as mean ± standard deviation and categorical variables as *n* (%)

*AF* atrial fibrillation, *CCI* Charlson Comorbidity Index and *OAC* oral anticoagulant

*P values for age from one-way ANOVA; all other P values from the chi-square test

### Statistical analyses

Categorical variables were presented as percentages, and continuous variables as mean ± standard deviation (SD). Differences in the incidence of registered comorbidities across education groups were assessed using the Chi-square test, and age was compared using one-way analysis of variance (ANOVA). Unadjusted Kaplan-Meier failure plots of the endpoint events across education groups were used. Cox regression models were used to compare cause-specific hazards of hospitalization across education groups for females and males separately. Because death precludes subsequent hospitalization for the study outcomes, these Cox models should be interpreted as cause-specific hazard models, with death treated as a censoring event. Results were reported as hazard ratios (HR) with 95% confidence intervals (CI). Analyses were performed unadjusted and adjusted for age, calendar year of AF diagnosis (1995–1999; 2000–2004; 2005–2008), AF as the primary or secondary diagnosis at the index hospitalization, and comorbidity burden using CCI comorbidity diagnoses and hypertension as binary variables. Age was modelled using restricted cubic splines with four knots, according to Harrell [26, 27]. We tested for non-proportionality using Schoenfeld residuals (STATA PHTEST). When a covariate violated the proportional hazards assumption, stratified Cox regression was used for that covariate, allowing the baseline hazard to vary across strata while retaining the estimated association between education level and the outcome. Variables handled by stratification are indicated in the respective tables. All analyses were performed using STATA versions 16 and 17 (StataCorp, College Station, TX, USA).

### Ethics

The study was conducted in accordance with the Declaration of Helsinki and approved by the Regional Ethical Review Board in Uppsala, Sweden (Dnr: 2009/273). Data were anonymized by the Swedish National Board of Health and Welfare and Statistics Sweden before delivery to the researchers, and the requirement for informed consent was waived.

## Results

**Fig. 1:**
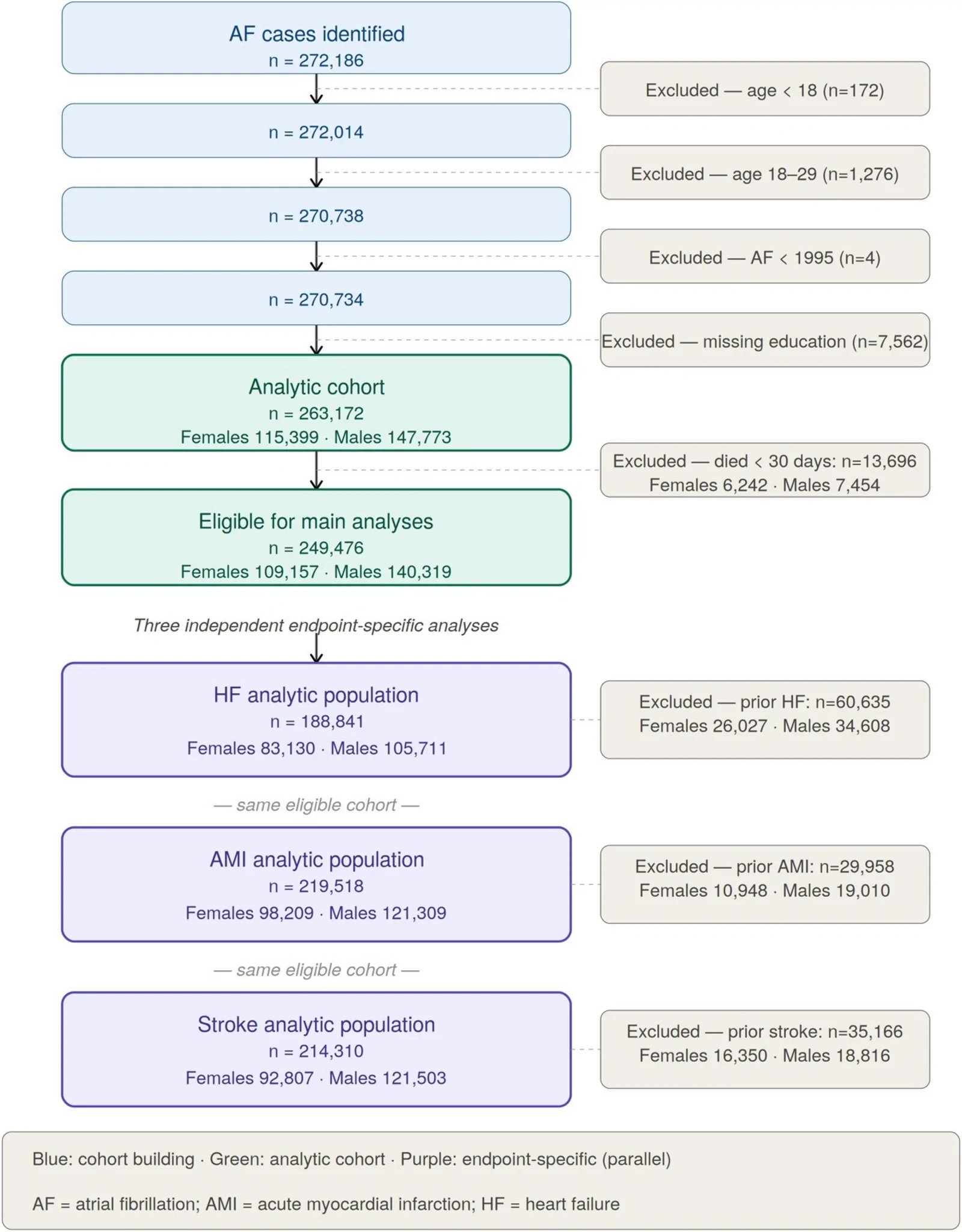
Flow diagram of cohort selection

Our database included 272,186 patients with a first recorded diagnosis of AF during hospitalization. Of these, 1,448 were excluded because of age < 30 years and 7,562 because data on education level were missing. Four patients were excluded because they were diagnosed with AF before 1995 (Fig. 1). The final study population comprised 263,172 individuals with a mean age of 72.5±10.4 years, 56.2% of whom were males.

The number of males and females was similar only in the primary education group. The proportion of females decreased as education level increased, comprising 37.8% in the secondary and 33.0% in the academic group. Among females, mean age at diagnosis was 76.8 ± 7.2, 72.1 ± 10.1, and 69.0 ± 11.1 years in the primary, secondary, and academic education groups, respectively. Among males, the corresponding ages were 73.3±9.2, 68.4±11.9, and 66.2±12.1 years (Table 1).

**Fig. 2:**
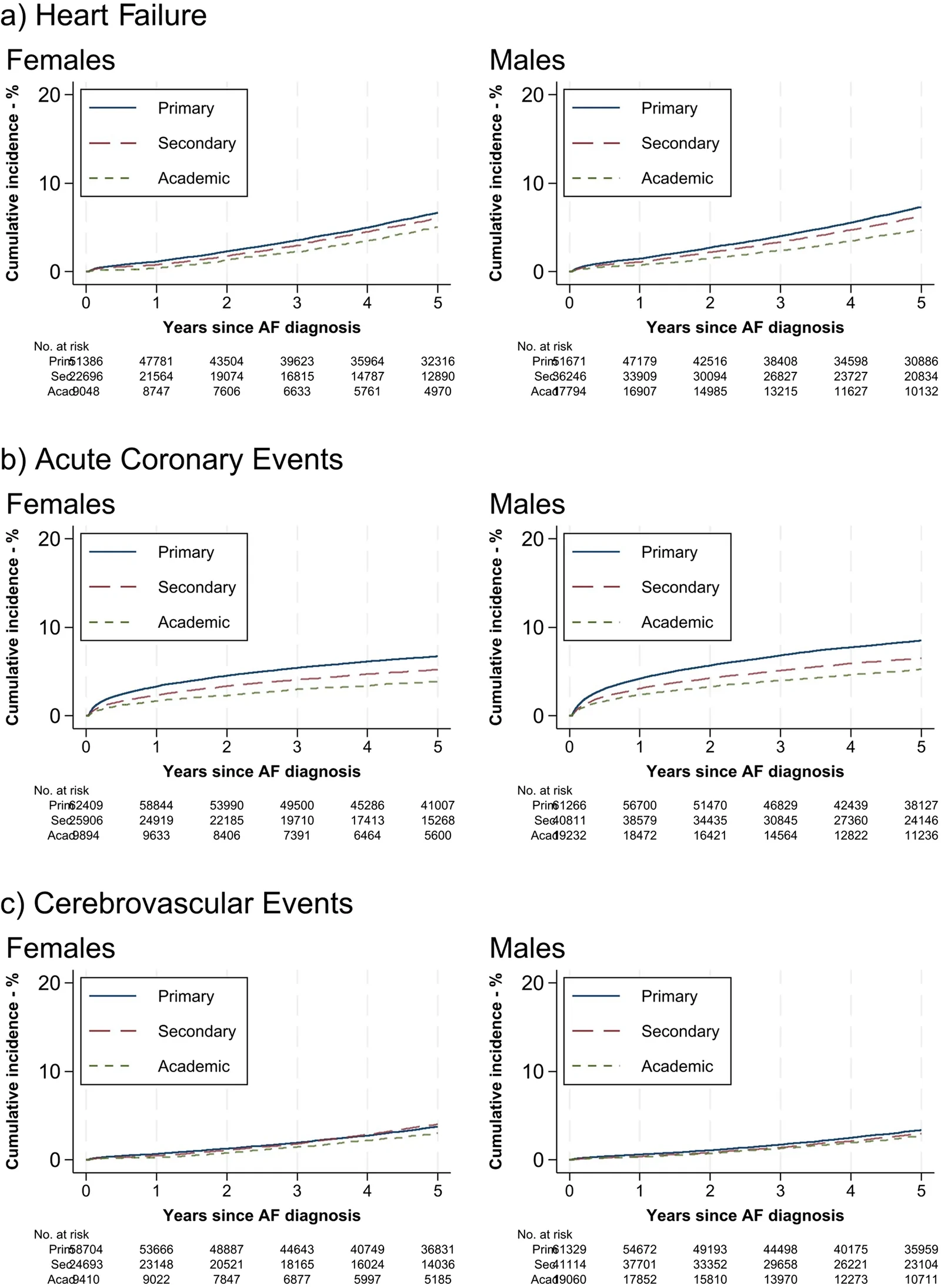
Kaplan-Meier failure estimates for (**a**) incident heart failure, (**b**) incident acute coronary events (AMI), and (**c**) incident cerebrovascular events (stroke), in females and males, respectively. Y-axis maximum is 20% in all panels. Abbreviations in the risk tables: P = Primary education; S = Secondary education; A = Academic education

Heart failure, AMI, or stroke occurred in 34,243, 25,908, and 21,708 patients during follow-up, respectively, accounting for 18.1%, 11.8%, and 10.1%, with the majority in the first year following diagnosis. Absolute event proportions decreased with increasing education level across all outcomes (Tables 2-4). All three outcomes were more common among patients with a lower education level (Fig. 2). HF and stroke were more common in females than in males across all education groups, whereas AMI was more common in males than in females across all education groups.

**Fig. 3:**
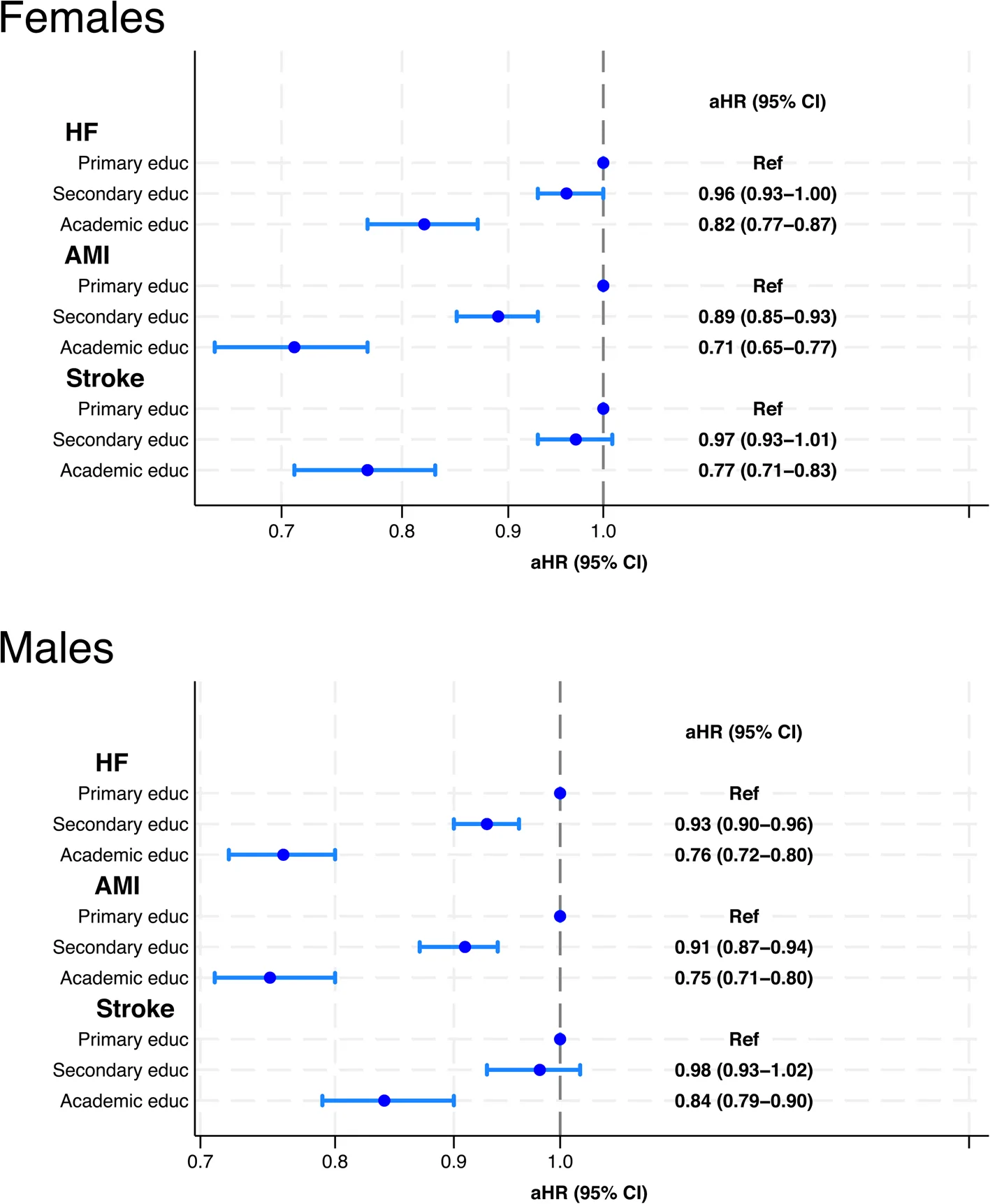
Forest plots of Hazard Ratios for risk of endpoint events by educational attainment in females and males, respectively

Higher education level was associated with a lower cause-specific hazard of HF and AMI (Fig. 3). For HF, adjusted HRs for secondary versus primary education were 0.96 (95% CI 0.93–1.00) in females and 0.93 (95% CI 0.90–0.96) in males; for academic versus primary education, HRs were 0.82 (95% CI 0.77–0.87) in females and 0.76 (95% CI 0.72–0.80) in males (Table 2). Sensitivity analyses including patients who died within 30 days following index hospitalization showed no difference in the results (Supplementary Tables 6 and 7).

**Table 2:** Five-year risk of heart failure by educational attainment using Cox regression

Females (*n* = 83 130)	*N﻿*	Events, *n* (%)	Rates *	Unadjusted HR (95% CI)	Adjusted † HR (95% CI)
*Education group*					
Primary	51 386	11 170 (21.7)	66.2	Ref	Ref
Secondary	22 696	3 748 (16.5)	48.0	0.73 (0.70–0.76)	0.96 (0.93–1.00)
Academic	9 048	1 059 (11.7)	32.7	0.50 (0.47–0.53)	0.82 (0.77–0.87)

Males (*n* = 105 711)	*N*	Events, *n* (%)	Rates*	Unadjusted HR (95% CI)	Adjusted † HR (95% CI)
*Education group*					
Primary	51 671	10 625 (20.6)	63.2	Ref	Ref
Secondary	36 246	5 614 (15.5)	44.7	0.71 (0.69–0.74)	0.93 (0.90–0.96)
Academic	17 794	2 027 (11.4)	31.2	0.50 (0.48–0.53)	0.76 (0.72–0.80)

*AF* atrial fibrillation, *CI* confidence interval, *HR* hazard ratio

*Crude rates are per 1 000 person-years

† Adjusted for age (restricted cubic splines); calendar period of AF diagnosis (1995–1999, 2000–2004, 2005–2008); AF as the primary or secondary admission diagnosis, and baseline comorbidities as defined in the dataset. Stratified Cox regression was used if non-proportional hazards were detected. Stratification was performed by earlier hypertension diagnosis, earlier myocardial infarction and calendar period of AF diagnoses in females; earlier myocardial infarction, earlier cerebrovascular event and age modelled as cubic splines in males

For AMI, adjusted HRs for secondary versus primary education were 0.89 (95% CI 0.85–0.93) in females and 0.91 (95% CI 0.87–0.94) in males; for academic versus primary education, adjusted HRs were 0.71 (95% CI 0.65–0.77) in females and 0.75 (95% CI 0.71–0.80) in males (Table 3). Sensitivity analyses showed similar results (Supplementary Tables 6 and 7).

**Table 3:** Five-year risk of acute myocardial infarction by educational attainment using Cox regression

Females (*n* = 98 209)	*N*	Events, *n* (%)	Rates *	Unadjusted HR (95% CI)	Adjusted † HR (95% CI)
*Education group*					
Primary	62 409	7 556 (12.1)	35.8	Ref	Ref
Secondary	25 906	2 278 (8.8)	24.8	0.70 (0.67–0.73)	0.89 (0.85–0.93)
Academic	9 894	577 (5.8)	15.8	0.45 (0.41–0.49)	0.71 (0.65–0.77)

Males (*n* = 121 309)	*N*	Events, *n* (%)	Rates *	Unadjusted HR (95% CI)	Adjusted † HR (95% CI)
*Education group*					
Primary	61 266	9 270 (15.1)	46.9	Ref	Ref
Secondary	40 811	4 607 (11.3)	32.6	0.70 (0.68–0.73)	0.91 (0.87–0.94)
Academic	19 232	1 620 (8.4)	23.0	0.50 (0.48–0.53)	0.75 (0.71–0.80)

*AF* atrial fibrillation, *CI* confidence interval, *HR* hazard ratio

*Crude rates are per 1 000 person-years

†Adjusted for age (restricted cubic splines), calendar period of AF diagnosis (1995–1999, 2000–2004, 2005–2008), AF as the primary or secondary admission diagnosis, and baseline comorbidities as defined in the dataset. Stratified Cox regression was used if non-proportional hazards were detected. Stratification was performed by earlier heart failure diagnosis, moderate or severe liver disease, AF as primary or secondary admission diagnosis and calendar period of AF diagnoses in females; earlier heart failure diagnosis, earlier diagnosis of diabetes, AF as primary or secondary admission diagnosis and calendar period of AF diagnoses in males

The association with stroke was significant only in the academic education group: adjusted HR 0.77 (95% CI 0.71–0.83) in females and 0.84 (95% CI 0.79–0.90) in males (Table 4). Sensitivity analyses including patients who died within 30 days after the index hospitalization showed hazard ratios being consistent in direction and magnitude with the main analyses. In females, the adjusted hazard ratios for secondary and academic education were 0.95 (95% CI 0.92–0.99) and 0.79 (95% CI 0.74–0.84) for heart failure, 0.87 (95% CI 0.83–0.91) and 0.67 (95% CI 0.62–0.73) for acute myocardial infarction, and 0.96 (95% CI 0.92–1.00) and 0.75 (95% CI 0.70–0.82) for stroke, respectively. In males, the corresponding adjusted hazard ratios were 0.92 (95% CI 0.89–0.95) and 0.75 (95% CI 0.72–0.79) for heart failure, 0.90 (95% CI 0.86–0.93) and 0.75 (95% CI 0.71–0.79) for acute myocardial infarction, and 0.98 (95% CI 0.93–1.02) and 0.85 (95% CI 0.79–0.90) for stroke, respectively (Supplementary Tables 6 and 7).

**Table 4:** Five-year risk of stroke by educational attainment using Cox regression

Females (*n* = 92 807)	*N*	Events, *n* (%)	Rates *	Unadjusted HR (95% CI)	Adjusted † HR (95% CI)
*Education group*					
Primary	58 704	8 165 (13.9)	41.4	Ref	Ref
Secondary	24 693	2 601 (10.5)	30.0	0.73 (0.70–0.76)	0.97 (0.93–1.01)
Academic	9 410	669 (7.1)	19.4	0.47 (0.44–0.51)	0.77 (0.71–0.83)

Males (*n* = 121 503)	*N*	Events, n (%)	Rates *	Unadjusted HR (95% CI)	Adjusted † HR (95% CI)
*Education group*					
Primary	61 329	6 001 (9.8)	29.4	Ref	Ref
Secondary	41 114	3 139 (7.6)	21.6	0.74 (0.71–0.77)	0.98 (0.93–1.02)
Academic	19 060	1 133 (5.9)	16.0	0.55 (0.52–0.59)	0.84 (0.79–0.90)

*AF* atrial fibrillation, *CI* confidence interval, *HR* hazard ratio

*Crude rates are per 1 000 person-years

†Adjusted for age (restricted cubic splines), calendar period of AF diagnosis (1995–1999, 2000–2004, 2005–2008), AF as the primary or secondary admission diagnosis, and baseline comorbidities as defined in the dataset. Stratified Cox regression was used if non-proportional hazards were detected. Stratification was performed by earlier myocardial infarction diagnosis, and calendar period of AF diagnoses in females; AF as primary or secondary admission diagnosis and calendar period of AF diagnoses in males

Across education groups, females had higher CHA₂DS₂-VA scores and CCI scores than males (Table 1). The proportion of females with very high thromboembolic risk (CHA_2_DS_2_-VA ≥ 4) was 53.5%, 38.6%, and 26.4% in the primary, secondary, and academic education groups, respectively, compared with 44.9%, 32.0%, and 24.4% among males. The proportion of patients with high comorbidity burden (CCI ≥ 5) was 67.6%, 52.2%, and 39.9% among females and 58.9%, 44.8%, and 34.7% among males in the primary, secondary, and academic education groups, respectively.

## Discussion

Education levels compared to the primary level were associated with lower cause-specific relative risk of subsequent incident HF and AMI, whereas the association with stroke was significant only in the academic education group.

The majority of HF, AMI, and stroke events occurred during the first year of follow-up. Although patients were defined by a first recorded AF diagnosis during hospitalization, this may not have represented their first-ever AF episode. It is reasonable to assume that a substantial proportion of our patients had incident or recent-onset AF, and that early initiation of treatment for AF and comorbidities may have contributed to improved risk factor control and delayed progression of AF and/or associated cardiovascular risk.

Heart failure, AMI, and stroke share modifiable cardiovascular risk factors, such as hypertension, smoking, and sedentary lifestyle, which are more prevalent among groups with lower education level [28]. These factors may have contributed to the observed associations between education level and morbidity. Differences in health literacy, access to care, and adherence to treatment recommendations may also have contributed, particularly for outcomes that are sensitive to risk factor control and secondary prevention [1]. These pathways were not directly measured in the present study.

Atrial fibrillation and HF share pathophysiological pathways involving atrial and ventricular remodelling, and the conditions may exert reciprocal effects on disease development and progression [29, 30]. The high incidence of HF observed in our cohort underscores the clinical importance of early identification and management of HF risk factors among patients with AF [29]. Trials of rhythm-control strategies in selected HF populations, including catheter ablation, suggest potential benefits in some patients; however, these findings may not be generalizable to an unselected nationwide AF population and do not explain the education gradient observed in our study [31, 32]. 

The association between AF and the cause-specific risk of incident AMI showed a similar pattern. Atrial fibrillation may be linked to AMI through shared risk factors such as hypertension, diabetes, and dyslipidaemia that promote systemic inflammation, atherosclerosis, and acute ischemic events. Tachyarrhythmia episodes may also cause myocardial ischemia and type 2 myocardial infarction and, rarely, coronary embolism during AF [33]. Conversely, AMI and coronary artery disease are associated with a higher risk of AF, creating a feed-forward loop that may increase morbidity [34]. This elevated risk was also observed in a retrospective observational study from Olmsted County, Minnesota that followed 2,768 patients with newly diagnosed AF for six years [35]. The 5-year unadjusted cumulative incidence of AMI was 14.5% in males and 14.3% in females, compared with 12.8% and 10.6% in our study, respectively. The higher incidence in the Olmsted study may partly reflect differences in endpoint definitions, as incident angina pectoris and unstable angina were also included. In that study, sex differences in incidence became more apparent after adjustment for age.

The association between education level and stroke risk in our study was weakened after adjustment for age and comorbidities, which may suggest that established clinical risk factors account for a larger proportion of the observed education gradient for stroke than for HF and AMI. This difference between outcomes may also reflect differences in preventive pathways. Stroke prevention in AF is strongly influenced by appropriate initiation and long-term adherence to oral anticoagulation, whereas prevention of HF and AMI depends more on control of cardiovascular risk factors, including hypertension, diabetes, dyslipidaemia, smoking, obesity, and physical inactivity. Socioeconomic differences may influence all three outcomes.

The role of anticoagulation is particularly relevant for stroke. A Finnish nationwide observational study of patients with incident AF reported socioeconomic differences in both initiation of oral anticoagulation therapy and treatment adherence, with an approximately 20% higher adherence in the highest compared to the lowest education group [36]. Differences in anticoagulation use and adherence could therefore contribute to stroke disparities. In our study, the treatment data were not available.

An Australian prospective observational cohort study reported a similar inverse relationship between education level and stroke risk in individuals with and without AF [37]. The authors described a non-linear association, with the lowest stroke risk observed in the highest education group. Interaction analyses indicated that lifestyle factors, psychological distress, and concomitant morbidity contributed substantially to the elevated risk. A Mendelian randomization study in populations of European ancestry also suggested an association between genetically predicted education level and stroke risk, with mediation through cardiovascular risk factors such as dyslipidaemia, hypertension, diabetes, and smoking [38]. Together, these findings support the interpretation that the weaker adjusted association with stroke in our cohort may reflect the combined effects of clinical risk factor burden, anticoagulation-related factors, and residual confounding.

Females had higher calculated stroke and comorbidity risk scores than males, which may be explained by their higher age at hospitalization [39, 40]. Females also had higher event rates of HF and stroke. These analyses were descriptive and since formal interaction testing was not performed, should not be interpreted as formal evidence of sex differences in the association between education level and cardiovascular morbidity among patients with AF. These findings align with a meta-analysis of sex differences in cardiovascular morbidity and mortality, which reported that AF confers a greater relative risk in females than in males [41]. 

From a clinical perspective, education level should be interpreted as a marker of social and behavioural risk rather than as a stand-alone clinical decision tool. Patients with lower education level may benefit from structured discharge information, closer follow-up after AF hospitalization, systematic assessment and treatment of modifiable cardiovascular risk factors, and support to improve adherence to prescribed therapies, including oral anticoagulation when indicated. It remains to be shown whether education level improves risk prediction beyond established clinical variables.

This large nationwide patient population provides statistical power and supports generalizability within similar healthcare systems. Because the cohort included patients with AF recorded during hospitalization in Sweden, the findings may not be generalizable to patients managed in outpatient care and primary care or to healthcare systems with different access, financing, or care pathways.

Among limitations to consider, the first diagnosis of AF in this study does not necessarily mean that it was the patient´s first-ever AF episode. Education level was used as a proxy for broader socioeconomic and behavioural factors and should not be interpreted as a causal exposure. We did not have information on smoking, BMI, lipid levels, blood pressure control, diet, physical activity, medication use, anticoagulation initiation, or treatment adherence during follow-up. These unmeasured factors may have contributed to the observed associations and may have resulted in residual confounding. This is particularly relevant for stroke, where the association with education level was attenuated after adjustment, suggesting that age and clinical risk factors may explain a substantial proportion of the observed gradient. Although sensitivity analyses including early deaths showed results consistent with the main analyses, this exclusion may have introduced some survivorship bias.

Because competing risk analyses were not performed, the Cox regression estimates should be interpreted as cause-specific hazard associations rather than estimates of absolute cumulative incidence in the presence of competing mortality.

## Conclusions

Among patients with a first detected AF during hospitalization, lower education level was associated with higher cause-specific risks of incident HF and AMI, whereas the association with stroke was weaker. Education level may be regarded as a complementary risk marker of future morbidity and may help to identify patients who could benefit from closer follow-up, systematic optimization of cardiovascular risk factors, and support for medication adherence.

## Supplementary Information

Supplementary Material 1: Supplementary Table 1. Definitions of atrial fibrillation and outcome events according to ICD-9 and ICD-10. Supplementary Table 2. Charlson comorbidity index comorbidities included in the statistical analysis. Supplementary Table 3. Comparison of five-year heart failure risk across education levels using Cox regression. Supplementary Table 4. Comparison of five-year risk of acute myocardial infarction across education levels based on Cox regression. Supplementary Table 5. Comparison of Five-year stroke risk across education levels based on Cox regression. Supplementary Table 6: Sensitivity analysis of five-year outcome risk by educational attainment using Cox regression, with and without a 30-day blanking period in females. Supplementary Table 7: Sensitivity analysis of five-year outcome risk relative to educational attainment based on Cox regression, with and without a 30-day blanking period in males.

## Data Availability

The data that support the findings of this study are available from the corresponding author upon reasonable request.

## Abbreviations

AF: Atrial Fibrillation
AMI: Acute Myocardial Infarction
CCI: Charlson Comorbidity Index
CHA_2_DS_2_-VA: Congestive heart failure, hypertension, age ≥ 75 years (2 points), diabetes mellitus, prior stroke/transient ischaemic attack/arterial thromboembolism (2 points), vascular disease, age 65–74 years (score)
CI: Confidence Interval
HF: Heart Failure
HR: Hazard Ratio
ICD: International Classification of Diseases
ISCED: International Standard Classification of Education
SD: Standard Deviation
SES: Socioeconomic Status
