# Supplementary material for: Educational level and cardiovascular outcomes in patients hospitalized with first-detected atrial fibrillation: a Swedish nationwide cohort study
Source: BMC Cardiovasc Disord. 2026 Jul 24;26:767. doi: 10.1186/s12872-026-06095-y (PMC13540872; doi:10.1186/s12872-026-06095-y)
Supplement: Supplementary file 1 [file 12872_2026_6095_MOESM1_ESM.docx]

**Supplementary Tables**

**Supplementary Table 1.** Definitions of atrial fibrillation and outcome events according to ICD-9 and ICD-10

| **Diagnosis** | **ICD-9** | **ICD-10** |
| --- | --- | --- |
| Atrial fibrillation | 427 D (DA, DB, DC, DD, DW) | I48 and I48.9 (A, B, C, D, E, F, P, X) |
| Myocardial infarction | 410 (A, B, W, X), 411 (A, B, C, X), 412 | I21 (0, 1, 2, 3, 4, 9), I22 (0, 1, 8, 9) I25.2 |
| Heart failure | 428 (A, B, X) | I50 (0, 1, 9) |
| Stroke | 433 (A, B, C, D, W, X), 434 (A, B, X), 435 | I63 (0, 1, 2, 3, 4, 5, 6, 8, 9), I64 and G45 (0, 1, 2, 3, 4, 8, 9) |

**Supplementary Table 2.** Charlson comorbidity index comorbidities included in the statistical analysis

| Diabetes mellitus | Chronic obstructive pulmonary disease |
| --- | --- |
| Acute myocardial infarction | Peripheral artery disease |
| Coronary artery disease | Dementia |
| Heart failure | Rheumatic disease |
| Cerebrovascular event | Mild liver disease |
| Cancer | Moderate to severe liver disease |
| Chronic kidney disease | Peptic ulcer |

**Supplementary Table 3.** Comparison of five-year heart failure risk across education levels using Cox regression

| **Females**  **(n=83 130)** | **n** | **Events (%)** | **Rates** | **Unadjusted**  **HR (95% CI)** | **Adjusted ^*^**†  **HR (95% CI)** |
| --- | --- | --- | --- | --- | --- |
| Education level |  |  |  |  |  |
| Primary | 51 386 | 11 170 (21.7) | 66.2 | Ref | Ref |
| Secondary | 22 696 | 3 748 (16.5) | 48.0 | 0.73 (0.70–0.76) | 0.96 (0.93–1.00) |
| Academic | 9 048 | 1 059 (11.7) | 32.7 | 0.50 (0.47–0.53) | 0.82 (0.77–0.87) |
| **Comorbidity** |  |  |  |  |  |
| Acute myocardial infarction |  |  |  |  | NA^†^ |
| Hypertension |  |  |  |  | NA^†^ |
| Diabetes |  |  |  |  | 1.57 (1.50–1.64) |
| Peripheral vascular disease |  |  |  |  | 1.57 (1.46–1.69) |
| Stroke |  |  |  |  | 0.84 (0.81–0.88) |
| Cancer |  |  |  |  | 1.09 (1.05–1.14) |
| Chronic renal failure |  |  |  |  | 1.77 (1.45 – 2.16) |
| Dementia |  |  |  |  | 0.91 (0.78 – 1.06) |
| Chronic pulmonary disease |  |  |  |  | 1.68 (1.59 – 1.77) |
| Rheumatic disease |  |  |  |  | 1.43 (1.34–1.53) |
| Mild liver disease |  |  |  |  | 1.14 (0.95–1.35) |
| Moderate or severe liver disease |  |  |  |  | 1.25 (0.74–2.12) |
| Hemiplegia/paraplegia |  |  |  |  | 1.17 (0.91–1.50) |
| Renal disease |  |  |  |  | 1.26 (1.06–1.50) |
| Peptic ulcer |  |  |  |  | 1.18 (1.06–1.30) |
| HIV |  |  |  |  | NA^‡^ |
| **Males**  **(n=105 711)** | **n** | **Events (%)** | **Rates** | **Unadjusted**  **HR (95% CI)** | **Adjusted ^*^**†  **HR (95% CI)** |
| Education level |  |  |  |  |  |
| Primary | 51 671 | 10 625 (20.6) | 63.2 | Ref | Ref |
| Secondary | 36 246 | 5 614 (15.5) | 44.7 | 0.71 (0.69–0.74) | 0.93 (0.90–0.96) |
| Academic | 17 794 | 2 027 (11.4) | 31.2 | 0.50 (0.48–0.53) | 0.76 (0.72 – 0.80) |
| **Comorbidity** |  |  |  |  |  |
| Acute myocardial infarction |  |  |  |  | NA† |
| Hypertension |  |  |  |  | 1.04 (1.01–1.08) |
| Diabetes |  |  |  |  | 1.52 (1.46–1.59) |
| Peripheral vascular disease |  |  |  |  | 1.50 (1.41–1.60) |
| Stroke |  |  |  |  | NA^†^ |
| Cancer |  |  |  |  | 1.14 (1.10–1.19) |
| Chronic renal failure |  |  |  |  | 1.28 (1.11–1.48) |
| Dementia |  |  |  |  | 0.88 (0.74–1.05) |
| Chronic pulmonary disease |  |  |  |  | 1.58 (1.50 – 1.65) |
| Rheumatic disease |  |  |  |  | 1.20 (1.10–1.31) |
| Mild liver disease |  |  |  |  | 1.44 (1.24–1.65) |
| Moderate or severe liver disease |  |  |  |  | 1.19 (0.80–1.78) |
| Hemiplegia/paraplegia |  |  |  |  | 0.86 (0.67–1.09) |
| Renal disease |  |  |  |  | 1.44 (1.27–1.63) |
| Peptic ulcer |  |  |  |  | 1.21 (1.12–1.31) |
| HIV |  |  |  |  | 3.06 (0.98 – 9.55) |
| * Adjusted for age (restricted cubic splines), year of AF diagnosis (1995–1999, 2000–2004, 2005–2008), and AF as the primary admission diagnosis. † The proportional hazards assumption was violated. Stratification was performed by earlier hypertension diagnosis, earlier myocardial infarction and calendar period of AF diagnoses in females; earlier myocardial infarction, earlier cerebrovascular event and age modelled as cubic splines in males.  ‡ HIV was not adjusted for because of the small number of patients with an HIV diagnosis. Crude rates are per 1 000 person-years. Abbreviations: HR, hazard ratio; CI, confidence interval. | | | | | |

**Supplementary Table 4.** Comparison of five-year risk of acute myocardial infarction across education levels based on Cox regression

| **Females**  **(n=98 209)** | **n** | **Events (%)** | **Rates** | **Unadjusted**  **HR (95% CI)** | **Adjusted ^*^**†  **HR (95% CI)** |
| --- | --- | --- | --- | --- | --- |
| Education level |  |  |  |  |  |
| Primary | 62 409 | 7 556 (12.1) | 35.8 | Ref | Ref |
| Secondary | 25 906 | 2 278 (8.8) | 24.8 | 0.70 (0.67 –0.73) | 0.89 (0.85 – 0.93) |
| Academic | 9 894 | 577 (5.8) | 15.8 | 0.45 (0.41 –0.49) | 0.71 (0.65 – 0.77) |
| **Comorbidity** |  |  |  |  |  |
| Heart failure |  |  |  |  | NA^†^ |
| Hypertension |  |  |  |  | 1.17 (1.1–1.22) |
| Diabetes |  |  |  |  | 1.64 (1.56–1.73) |
| Peripheral vascular disease |  |  |  |  | 2.01 (1.86–2.18) |
| Stroke |  |  |  |  | 1.06 (0.99–1.11) |
| Cancer |  |  |  |  | 1.01 (0.96–1.07) |
| Chronic renal failure |  |  |  |  | 1.26 (1.03–1.56) |
| Dementia |  |  |  |  | 0.89 (0.74–1.07) |
| Chronic pulmonary disease |  |  |  |  | 1.47 (1.38–1.56) |
| Rheumatic disease |  |  |  |  | 1.35 (1.25–1.47) |
| Mild liver disease |  |  |  |  | 1.27 (1.05 – 1.55) |
| Moderate or severe liver disease |  |  |  |  | NA^†^ |
| Hemiplegia/paraplegia |  |  |  |  | 1.45 (1.10–1.91) |
| Renal disease |  |  |  |  | 1.72 (1.45–2.05) |
| Peptic ulcer |  |  |  |  | 1.15 (1.02–1.30) |
| HIV |  |  |  |  | NA^‡^ |
| **Males**  **(n=121 309)** | **n** | **Events (%)** | **Rates** | **Unadjusted**  **HR (95% CI)** | **Adjusted ^*^**†  **HR (95% CI)** |
| Education level |  |  |  |  |  |
| Primary | 61 266 | 9 270 (15.1) | 46.9 | Ref | Ref |
| Secondary | 40 811 | 4 607 (11.3) | 32.6 | 0.70 (0.68 –0.73) | 0.91 (0.87 – 0.94) |
| Academic | 19 232 | 1 620 (8.4) | 23.0 | 0.50 (0.48 – 0.53) | 0.75 (0.71 – 0.80) |
| **Comorbidity** |  |  |  |  |  |
| Heart failure |  |  |  |  | NA^†^ |
| Hypertension |  |  |  |  | 1.08 (1.04–1.12) |
| Diabetes |  |  |  |  | NA^†^ |
| Peripheral vascular disease |  |  |  |  | 1.93 (1.82–2.04) |
| Stroke |  |  |  |  | 1.12 (1.07–1.17) |
| Cancer |  |  |  |  | 1.00 (0.95–1.04) |
| Chronic renal failure |  |  |  |  | 1.32 (1.16–1.52) |
| Dementia |  |  |  |  | 0.81 (0.67–0.98) |
| Chronic pulmonary disease |  |  |  |  | 1.25 (1.19–1.32) |
| Rheumatic disease |  |  |  |  | 1.25 (1.14–1.37) |
| Mild liver disease |  |  |  |  | 1.29 (1.11–1.49) |
| Moderate or severe liver disease |  |  |  |  | 1.36 (0.93–1.98) |
| Hemiplegia/paraplegia |  |  |  |  | 1.11 (0.89–1.40) |
| Renal disease |  |  |  |  | 1.51 (1.33–1.70) |
| Peptic ulcer |  |  |  |  | 1.10 (1.01–1.20) |
| HIV |  |  |  |  | 3.06 (0.76–12.2) |
| * Adjusted for age (restricted cubic splines), year of AF diagnosis (1995–1999, 2000–2004, 2005–2008), and AF as the primary admission diagnosis. † The proportional hazards assumption was violated. Stratification was performed by earlier heart failure diagnosis, moderate or severe liver disease, AF as primary or secondary admission diagnosis and calendar period of AF diagnoses in females; earlier heart failure diagnosis, earlier diagnosis of diabetes, AF as primary or secondary admission diagnosis and calendar period of AF diagnoses in males.  ‡ HIV was not adjusted for because of few patients with an HIV diagnosis. Crude rates are per 1 000 person-years.  Abbreviations: HR, hazard ratio; CI, confidence interval. | | | | | |

**Supplementary Table 5.** Comparison of Five-year stroke risk across education levels based on Cox regression

| **Females**  **(n=92 807)** | **n** | **Events (%)** | **Rates** | **Unadjusted**  **HR (95% CI)** | **Adjusted ^*^**†  **HR (95% CI)** |
| --- | --- | --- | --- | --- | --- |
| Education level |  |  |  |  |  |
| Primary | 58 704 | 8 165 (13.9) | 41.4 | Ref | Ref |
| Secondary | 24 693 | 2 601 (10.5) | 30.0 | 0.73 (0.70–0.76) | 0.97 (0.93–1.01) |
| Academic | 9 410 | 669 (7.1) | 19.4 | 0.47 (0.44–0.51) | 0.77 (0.71–0.83) |
| **Comorbidity** |  |  |  |  |  |
| Acute myocardial infarction |  |  |  |  | NA^†^ |
| Heart failure |  |  |  |  | 0.98 (0.94–1.03) |
| Hypertension |  |  |  |  | 1.14 (1.09–1.19) |
| Diabetes |  |  |  |  | 1.32 (1.25–1.39) |
| Peripheral vascular disease |  |  |  |  | 1.34 (1.23–1.47) |
| Cancer |  |  |  |  | 0.96 (0.91 – 1.01) |
| Chronic renal failure |  |  |  |  | 0.88 (0.69 – 1.14) |
| Dementia |  |  |  |  | 1.03 (0.86 – 1.23) |
| Chronic pulmonary disease |  |  |  |  | 0.85 (0.79 – 0.92) |
| Rheumatic disease |  |  |  |  | 1.01 (0.92–1.10) |
| Mild liver disease |  |  |  |  | 1.24 (1.02–1.51) |
| Moderate or severe liver disease |  |  |  |  | 1.56 (0.88–2.75) |
| Hemiplegia/paraplegia |  |  |  |  | 1.49 (1.10–2.01) |
| Renal disease |  |  |  |  | 1.36 (1.11–1.66) |
| Peptic ulcer |  |  |  |  | 1.19 (1.06–1.34) |
| HIV |  |  |  |  | NA^‡^ |
| **Males**  **(n=121 503)** | **n** | **Events (%)** | **Rates** | **Unadjusted**  **HR (95% CI)** | **Adjusted ^*^**†  **HR (95% CI)** |
| Education level |  |  |  |  |  |
| Primary | 61 329 | 6 001 (9.8) | 29.4 | Ref | Ref |
| Secondary | 41 114 | 3 139 (7.6) | 21.6 | 0.74 (0.71–0.77) | 0.98 (0.93–1.02) |
| Academic | 19 060 | 1 133 (5.9) | 16.0 | 0.55 (0.52–0.59) | 0.84 (0.79–0.90) |
| **Comorbidity** |  |  |  |  |  |
| Acute myocardial infarction |  |  |  |  | 1.07 (1.01–1.13) |
| Heart failure |  |  |  |  | 0.94 (0.90–0.99) |
| Hypertension |  |  |  |  | 1.21 (1.15–1.27) |
| Diabetes |  |  |  |  | 1.48 (1.40–1.55) |
| Peripheral vascular disease |  |  |  |  | 1.29 (1.18–1.40) |
| Cancer |  |  |  |  | 0.95 (0.90–1.01) |
| Chronic renal failure |  |  |  |  | 1.13 (0.93–1.37) |
| Dementia |  |  |  |  | 1.15 (0.9 –1.43) |
| Chronic pulmonary disease |  |  |  |  | 0.94 (0.87–1.01) |
| Rheumatic disease |  |  |  |  | 1.17 (1.04–1.32) |
| Mild liver disease |  |  |  |  | 1.17 (0.96–1.44) |
| Moderate or severe liver disease |  |  |  |  | 1.10 (0.62–1.94) |
| Hemiplegia/paraplegia |  |  |  |  | 1.95 (1.51–2.16) |
| Renal disease |  |  |  |  | 1.02 (0.86–1.22) |
| Peptic ulcer |  |  |  |  | 1.14 (1.03–1.26) |
| HIV |  |  |  |  | NA^‡^ |
| * Adjusted for age (restricted cubic splines), year of AF diagnosis (1995–1999, 2000–2004, 2005–2008), and AF as the primary admission diagnosis. † The proportional hazards assumption was violated. Stratification was performed by earlier myocardial infarction diagnosis, and calendar period of AF diagnoses in females; AF as primary or secondary admission diagnosis and calendar period of AF diagnoses in males. ‡ HIV was not adjusted for because of few patients with an HIV diagnosis. Crude rates are per 1 000 person-years. Abbreviations: HR, hazard ratio; CI, confidence interval. | | | | | |

**Supplementary Table 6:** Sensitivity analysis of five-year outcome risk by educational attainment using Cox regression, with and without a 30-day blanking period in females

| **Without a 30-day blanking period** | **Adjusted ***  **HR (95% CI) for HF** | **Adjusted ***  **HR (95% CI) for AMI** | **Adjusted ***  **HR (95% CI) for Stroke** |
| --- | --- | --- | --- |
| Primary education | Ref | Ref | Ref |
| Secondary education | 0.95 (0.92–0.99) | 0.87 (0.83–0.91) | 0.96 (0.92–1.00) |
| Academic education | 0.79 (0.74–0.84) | 0.67 (0.62–0.73) | 0.75 (0.70–0.82) |
| *Adjusted for age as spline, year of AF diagnosis (1995–1999, 2000–2004, 2005–2008), AF as main admission diagnosis and CCI score (0, 1, 2, 3, 4, 5, 6, 7, ≥8).  Stratified Cox regression was used if non-proportional hazards.  Crude rates per 1 000 person-years; HR Hazard ratios; CI Confidence intervals. | | | |

**Supplementary Table 7**: Sensitivity analysis of five-year outcome risk relative to educational attainment based on Cox regression, with and without a 30-day blanking period in males

| **Without a 30-day blanking period** | **Adjusted ***  **HR (95% CI) for HF** | **Adjusted ***  **HR (95% CI) for AMI** | **Adjusted ***  **HR (95% CI) for Stroke** |
| --- | --- | --- | --- |
| Primary education | Ref | Ref | Ref |
| Secondary education | 0.92 (0.89–0.95) | 0.90 (0.86–0.93) | 0.98 (0.93–1.02) |
| Academic education | 0.75 (0.72–0.79) | 0.75 (0.71–0.79) | 0.85 (0.79–0.90) |
| *Adjusted for age as spline, year of AF diagnosis (1995–1999, 2000–2004, 2005–2008), AF as main admission diagnosis and CCI score (0, 1, 2, 3, 4, 5, 6, 7, ≥8).  Stratified Cox regression was used if non-proportional hazards.  Crude rates per 1 000 person-years; HR Hazard ratios; CI Confidence intervals. | | | |
